# The Dual Role of Cellular Senescence in Macrophages: Unveiling the Hidden Driver of Age-Related Inflammation in Kidney Disease

**DOI:** 10.7150/ijbs.104404

**Published:** 2025-01-01

**Authors:** Yi-bing Wang, Tong Li, Feng-yu Wang, Xin Yao, Qiu-xiang Bai, Hong-wei Su, Jian Liu, Li Wang, Rui-zhi Tan

**Affiliations:** 1 Department of Radiology, the Affiliated Hospital, Southwest Medical University, 646000 Luzhou, China.; 2 Research Center of Integrated Traditional Chinese and Western Medicine, the Affiliated Traditional Chinese Medicine Hospital, Southwest Medical University, 646000 Luzhou, China.; 3 College of Integration of Traditional Chinese and Western Medicine, Southwest Medical University, 646000 Luzhou, China.; 4 Department of Medical Imaging, Southwest Medical University, 646000 Luzhou, China.; 5 Department of Anesthesiology, Southwest Medical University, 646000 Luzhou, China.; 6 Department of Urology, the Affiliated Traditional Chinese Medicine Hospital, Southwest Medical University, 646000 Luzhou, China.; 7 Department of Nephrology, the Affiliated Traditional Chinese Medicine Hospital, Southwest Medical University, 646000 Luzhou, China.

**Keywords:** senescence, macrophage, kidney, senescence-associated secretory phenotype, inflammation

## Abstract

Aging is a complex biological process that involves the gradual decline of cellular, tissue, and organ functions. In kidney, aging manifests as tubular atrophy, glomerulosclerosis, and progressive renal function decline. The critical role of senescence-associated macrophage in diseases, particularly kidney diseases, is increasingly recognized. During this process, macrophages exhibit a range of pro-damage response to senescent tissues and cells, while the aging of macrophages themselves also significantly influences disease progression, creating a bidirectional regulatory role between aging and macrophages. To explore this bidirectional mechanism, this review will elucidate the origin, characteristic, phenotype, and function of macrophages in response to the senescence-associated secretory phenotype (SASP), extracellular vesicles from senescent cells, and the senescence cell-engulfment suppression (SCES), particularly in the context of kidney disease. Additionally, it will discuss the characteristics of senescent macrophage, such as common markers, and changes in autophagy, metabolism, gene regulation, phagocytosis, antigen presentation, and exosome secretion, along with their physiological and pathological impacts on renal tissue cells. Furthermore, exploring therapies and drugs that modulate the function of senescent macrophages or eliminate senescent cells may help slow the progression of kidney aging and damage.

## 1. Introduction

Aging is a complex biological process that profoundly affects tissues and organs throughout the body^1^. As one ages, there is a gradual decline in tissue function, reduced cellular regenerative capacity, and less efficient repair processes compared to those in youth. With the progression of aging, cellular functions gradually deteriorate. The ability of cells to divide diminishes, telomeres shorten, DNA damage accumulates, and processes such as autophagy and apoptosis may be activated, all contributing to a decline in the regenerative and reparative abilities of tissues^2^. There is a decrease in the synthesis of collagen and other structural proteins in the extracellular matrix, accompanied by an increase in degradation. This results in reduced elasticity and strength of tissues, thereby affecting the overall function of organs. For instance, the skin loses its elasticity and tension, bones become fragile, and blood vessels harden^3^-^5^. More importantly, aging is often accompanied by a chronic low-grade inflammatory state known as “inflammaging”^6^. This chronic inflammation may lead to tissue damage and further deterioration of organ function. Additionally, the decline in immune function and the surge in oxidative stress associated with aging also impact tissue damage, aging, and repair^7^, ^8^.

Notably, aging is one of the primary factors contributing to the gradual decline in kidney function^9^. With advancing age, the glomerular filtration rate (GFR) gradually decreases, leading to diminished renal clearance capacity. This functional decline increases the risk of chronic kidney disease (CKD). Moreover, age-related structural changes in the kidney, such as glomerulosclerosis, interstitial fibrosis, and tubular atrophy, further heighten the likelihood of kidney disease. Significantly, aging-induced impairments in the kidney's ability to metabolize drugs, regulate blood pressure, and manage cardiovascular events exacerbate the onset and progression of kidney disease^10^.

Macrophages are pivotal cells within the immune system, playing multifaceted roles in the body's immune defense mechanisms. The fundamental function of macrophages is to phagocytose and digest foreign pathogens (such as bacteria, viruses, and fungi), as well as dead cells and debris within the body - a process known as phagocytosis. By engulfing these materials, macrophages prevent their spread and the potential for infection^11^. In addition to phagocytosing pathogens, macrophages can break them down into small fragments and present these antigenic fragments to T cells, thereby activating the adaptive immune response^12^. This process of antigen presentation is crucial for initiating specific immune responses. Furthermore, under various physiological and pathological conditions, macrophages secrete a variety of cytokines that regulate inflammatory responses and promote the activation and migration of other immune cells. After the resolution of inflammation and infection, macrophages also participate in tissue repair and regeneration. They can clear remnants of damaged tissue and secrete factors that promote healing, aiding in tissue recovery^13^, ^14^. However, in the context of aging, studies have reported that senescent cells and tissues influence macrophages primarily through three mechanisms: the senescence-associated secretory phenotype (SASP)^15^, extracellular vesicles (EVs)^16^, and senescence cell-mediated phagocytosis suppression (SCES)^17^. These senescence phenotypes can enhance the pro-damage and inflammatory responses of macrophages, thereby accelerating disease progression.

Recent studies suggest that macrophages in aging kidneys may be key regulatory cells that promote age-related kidney damage^18^-^20^. While extensive research has documented the function of macrophages in aging kidneys, a systematic review of how senescent tissue cells, particularly in the kidneys, affect macrophages and the underlying mechanisms remains lacking. Interestingly, beyond the physiological and pathological effects of senescent tissue cells on macrophages, conversely, senescent macrophages also exert significant pathophysiological regulatory effects on tissue cells, forming a bidirectional regulatory model between aging and macrophages. In this context, the present article will systematically elucidate the origin, characteristics, and functions of macrophages, as well as their responses to aging tissue cells, particularly in the kidneys, and their subsequent role in modulating disease progression. Furthermore, we will discuss the role of senescent macrophages in promoting inflammation and fibrosis in kidney tissues, clarifying the bidirectional regulatory effects of aging and macrophages in kidney disease. Additionally, we will explore therapeutic applications targeting the mechanisms associated with senescent macrophages.

## 2. Macrophages and Senescence

### 2.1 Origin of macrophages

#### 2.1.1 Tissue-resident macrophages

Macrophages are immune cells in the human body that are widely distributed and highly diverse, initially proposed by Elie Metchnikoff. Due to their complex origins, macrophages can be classified into tissue-resident macrophages and infiltrating macrophages. Based on their functional plasticity mediated by microenvironmental signals, they can also be categorized as tumor-associated macrophages. Additionally, due to their functional roles in tissue injury and repair, macrophages can be further divided into pro-inflammatory and anti-inflammatory macrophages, among others^21^. Given the complexity of macrophage types, understanding their origins is crucial for comprehending their functions across various tissues.

**Figure 1:**
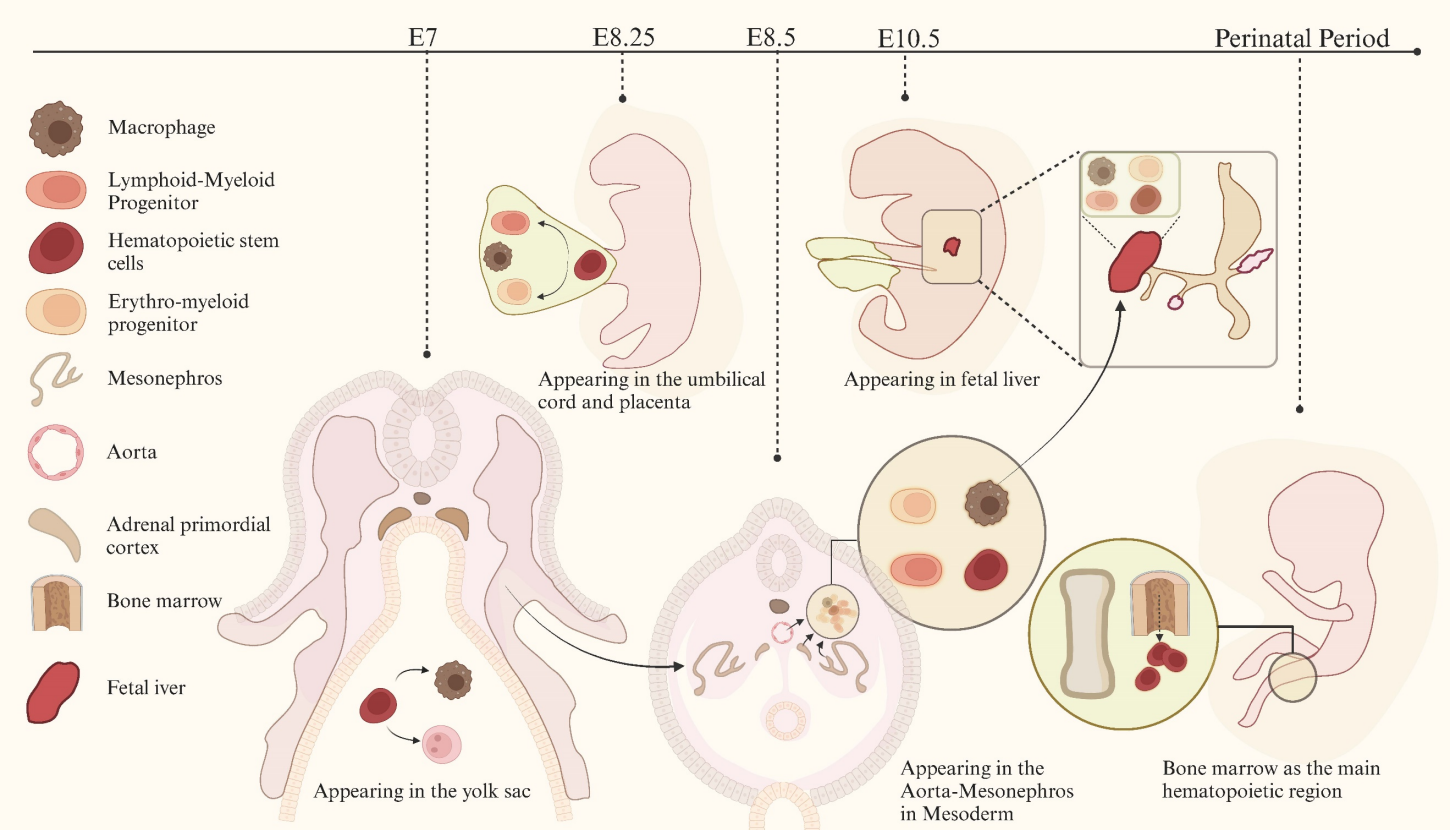
Development of macrophages.

In mammals, macrophages are first observed in early pregnancy (embryonic day E6.5 to E8.5), proliferating in the yolk sac of the extraembryonic ectoderm^22^, ^23^. These macrophages differentiate and mature directly in the yolk sac without going through a monocyte intermediate stage. Subsequent studies on the developmental sequence of hematopoietic cells^23^-^30^ suggest that macrophages originate from the yolk sac, fetal liver, and bone marrow at different stages of individual development^31^-^33^. Past research has shown that tissue-resident macrophages originate from embryonic precursors in the yolk sac and fetal liver, developing alongside their resident tissues during embryogenesis. They persist into adulthood and are maintained independently of blood monocyte replenishment^34^, ^35^. The development of macrophages is shown in detail in Figure 1. Although numerous studies have indicated that yolk sac-derived macrophages in various tissues may undergo progressive replacement during development, resulting in a reduction in their proportion, a subset of these cells persists and can become the primary targets for amplification in specific pathological states^22^, ^36^.

#### 2.1.2 Monocyte-derived macrophages

In order to purify bone marrow progenitor cells, Stephen J. Jenkins and David A. Hume proposed a model based on the use of Flt3 (CD135) and Csf1r (CD115) as key markers^37^, ^38^: Hematopoietic stem cells undergo differentiation to form lymphoid-sensitized multipotent progenitors (LMPP), which subsequently differentiate into granulocyte-macrophage progenitors (GMP), and then dendritic progenitors (CDP). This differentiation process culminates in the generation of macrophages and dendritic cell precursors (MDP), whereas the absence of Flt3 results in the production of monocyte progenitors (MoP). Conversely, MDPs that are not deficient in Flt3 differentiate into precursor dendritic cells (pre-DC). Following the generation of MoP, differentiation is controlled by CSF1, which initially differentiates to produce Ly6C+ cells. Subsequently, a proportion of the Ly6C+ cells migrate into the tissues and differentiate into macrophages, including those found in the blood^34^ and the intestine^39^, while another proportion differentiate into Ly6C- cells. The Ly6C- monocytes represent a terminal stage of differentiation.

Other studies have also indicated that monocyte progenitors develop into late-proliferative transitional pre-monocyte precursors (TpMo) before further differentiating into Ly6Clo monocytes, which subsequently enter the circulation and differentiate into mature Ly6Clo monocytes^40^, ^41^. Subsequently, these monocytes migrate to various tissues, where they differentiate into macrophages. It is worthy of note that upon recruitment to inflammatory tissues, monocytes do not immediately differentiate into macrophages. Rather, they may undergo self-renewal within the tissue while maintaining a monocyte identity^42^.

#### 2.1.3 Macrophage polarization into M1-type and M2 macrophages

Macrophages exhibit different morphologies, phenotypes and functions under the influence of different environmental signals, a phenomenon known as macrophage polarization. Macrophages are primarily polarized into two types: classically activated (M1) and alternatively activated (M2) macrophages. M1 macrophages typically appear in Th1 immune responses^33^, and are activated by interferon-γ (IFN-γ), lipopolysaccharide (LPS), granulocyte-macrophage colony-stimulating factor (GM-CSF) and toll-like receptors (TLRs), leading to increased secretion of cytokines such as IL-1β, TNF, IL-12 and IL-18. They express high levels of markers such as major histocompatibility complex class II (MHC-II), CD60, CD68, CD80 and CD86, and have potent pro-inflammatory and anti-proliferative effects. M2 macrophages normally arise in Th2 immune responses^33^, and are induced by CSF-1, IL-4, IL-10, transforming growth factor beta (TGF-β), IL-13 and stimuli from fungal and helminth infections. They secrete IL-10, TGF-β, insulin-like growth factor 1, vascular endothelial growth factor (VEGF), epidermal growth factor (EGF), platelet-derived growth factor (PDGF) and other cytokines, and show increased cell surface expression of arginase1 (Arg1) and markers such as CD163, CD204 and the mannose receptor (CD206)^43^-^46^. Based on this, M2 macrophages are further subdivided into different types according to stimulation by different factors. For example, M2a is induced by IL-4/IL-13, M2b by immune complexes and Toll-like receptor or IL-1R agonists, M2c by IL-10 and M2d by IL-6 or adenosine^47^, ^48^.

M1 macrophages are known to promote inflammatory responses, while M2 macrophages are involved in tissue repair and healing. An imbalance between M1 and M2 macrophages can lead to disease. Since this polarization is reversible, i.e., M1 and M2 macrophages can be converted to each other through pathway regulation, modulation of signaling pathways such as JAK/STATs, TGF-β, and PPARγ, as well as metabolism, can achieve interconversion of M1 and M2 macrophages^47^. Therefore, targeting the balance between M1 and M2 macrophages may ameliorate diseases. For example, quercetin has been shown to suppress M1 macrophage polarization by downregulating the activity of NF-κB p65 and IRF5, thereby reducing the expression of M1 macrophage markers such as iNOS and IL-12. Quercetin also inhibits M2 macrophage polarization, decreases their proportion in the kidney, and decreases the expression of M2 macrophage markers such as Arg-1 and IL-10, thereby adjusting the proportion of macrophage polarization types and alleviating renal inflammation, injury, and fibrosis processes^49^. In the context of clear cell renal cell carcinoma (ccRCC), a prognostic model study indicated that TRAF2 promotes the malignant progression of ccRCC by regulating macrophage polarization, migration, and angiogenesis. Consequently, TRAF2 has been identified as a potential novel therapeutic target for advanced ccRCC^50^.

#### 2.1.4 Specialized macrophages in the tumor microenvironment: tumor-associated macrophages

Tumor-associated macrophages (TAMs) are macrophages that are recruited and infiltrate tumor tissue by tumor-derived chemotactic factors such as macrophage colony-stimulating factor (M-CSF), CCL2, and CCL5^51^. The majority of TAMs are derived from the transformation of macrophages derived from circulating monocytes, with a smaller proportion derived from tissue-resident macrophages^52^ and monocyte-derived myeloid suppressor cells (M-MDSCs)^53^. The M2d subpopulation, which forms after macrophage polarization, is a major component of TAMs^54^. TAMs exhibit a polarized phenotype with characteristics of both M1 and M2 macrophages. M1-type macrophages are involved in pro-inflammatory responses and tumor growth inhibition, whereas M2-type macrophages are involved in anti-inflammatory processes and tumor growth promotion. In addition, TAMs are involved in angiogenesis, metabolic reprogramming, and regulation of the immune microenvironment. Therefore, TAMs may serve as a therapeutic target for anti-tumor treatments^55^-^57^.

Previous studies have implicated signaling pathways and cytokines such as CCL8, CCL2/CCR2, CSF-1/CSF-1R, STAT3, STAT6, MMPs, AMPK, TLR3, and SIRPα in the regulation of TAMs and tumor progression^58^-^61^. Recent research has shown that SLC3A2 regulates arachidonic acid through metabolic reprogramming, which can promote the polarization of TAMs toward the M2 phenotype in lung adenocarcinoma. Inhibition of its expression can limit tumor proliferation and metastasis^62^. JMJD6 regulates TAM polarization in lung tumors through the STAT3/IL-10 axis^63^. In breast cancer, TAMs respond to a stiff fibrotic tumor microenvironment by initiating a TGF-β-directed collagen biosynthesis program and further impair CD8+ T cell function by altering amino acids, thereby modifying the immune microenvironment^64^. In a renal transcriptome atlas, FN1+ TAMs were found to express high levels of FN1 and the scavenger receptor MARCO, identifying them as a specific macrophage subpopulation in renal cancer^65^. Single-cell sequencing revealed that MHC-II TAMs, which highly express HLA-DRB5, APOE, and APOC1, are enriched in the tumor core, and FN1+ TAMs may be pro-tumorigenic in renal cell carcinoma (RCC)^66^. Therefore, targeting TAM-related mechanisms in RCC may provide new therapeutic strategies. For example, an experiment showed that FOXK1 upregulation was associated with TAM infiltration, and its knockdown inhibited the Wnt signaling pathway, suggesting that FOXK1 could be a potential therapeutic target for RCC^67^.

### 2.2 Effects of senescent cells and tissues on macrophages

Senescent cells are cells that have permanently exited the cell cycle due to various factors such as DNA damage and oxidative stress^68^. Although they no longer divide, they remain metabolically active and secrete a variety of cytokines, chemokines, and proteases, collectively referred to as the SASP. There is increasing evidence that senescent cells and tissues influence macrophage function and polarization through multiple mechanisms, thereby influencing tissue inflammation, repair, and homeostasis. This interaction becomes particularly important during aging, with implications for health and disease progression^69^. Current research suggests that the influence of senescent cells and tissues on macrophages occurs primarily through three mechanisms: SASP, EVs, and the recently proposed SCES. These influences include a variety of pro- and anti-inflammatory mechanisms, macrophage polarization, phagocytic activity, migration and recruitment, and differentiation into specific cell types.

#### 2.2.1 Phenotypic pathways of senescent cell secretion in different tissues

**Figure 2:**
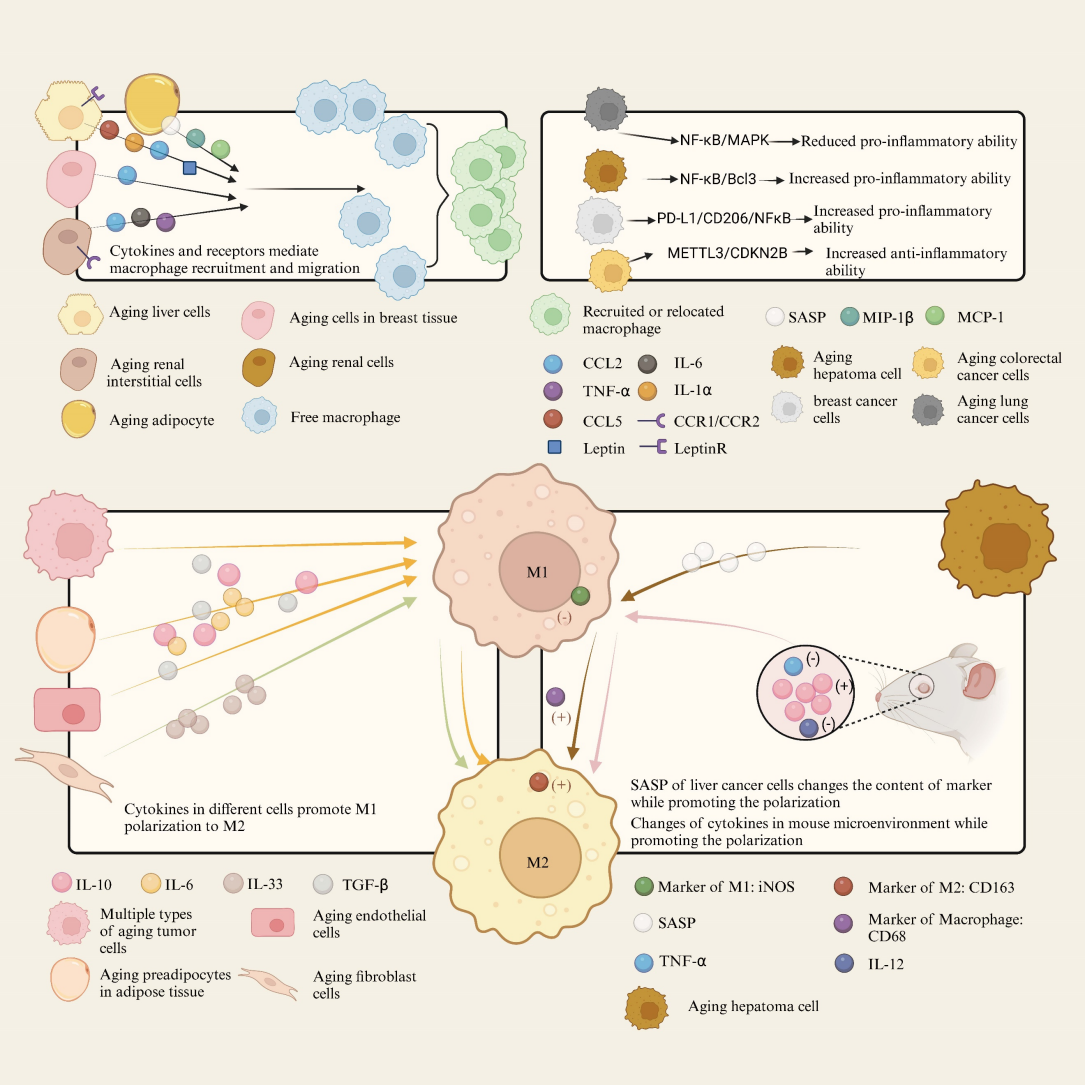
SASP-related mechanism of senescent cells and tissues acting on macrophage.

The SASP is characterized by the abundant secretion of inflammatory cytokines, chemokines, growth factors and proteases by senescent cells. These factors significantly alter the tissue microenvironment and thereby influence macrophage behavior. Macrophages are attracted by SASP factors to remove senescent cells^70^, but they can also be affected by the presence of senescent cells^71^. The SASP-related mechanism of senescent cells and tissues acting on macrophage is shown in Figure 2.

##### 2.2.1.1 SASP affects macrophage recruitment and migration

Senescent hepatocytes directly secrete cytokines such as IL-1α, Leptin R, leptin, CCL2, and CCL5, which can recruit immune cells such as CD4+ T cells and macrophages^72^. In the kidney, senescent CD73-expressing mesenchymal stromal cells (kMSCs) recruit monocyte-derived Ly6C+CCR2+ macrophages, which express pro-inflammatory cytokines, through cytokines such as CCL2, CCL5, and IL-1α^73^. Compared to mice with the P72 variant, mice with the R72 variant have more senescent cells in their mammary tissue. The accumulation of these senescent cells promotes the formation of the SASP with a marked increase in CCL2 levels. CCL2 is a key chemokine responsible for recruiting macrophages to sites of inflammation or tumors. Recruited macrophages not only enhance the inflammatory response in tissues, but can also promote tumor angiogenesis by secreting factors such as VEGFA, further supporting tumor growth. In addition, these macrophages may create a positive feedback loop that exacerbates the SASP state of the tissue by secreting more inflammatory and pro-angiogenic factors^74^.

SASP also affects macrophage migration. In the liver, SASP promotes the migration of inflammatory (GM-CSF-derived) rather than non-inflammatory (CSF-1-derived) human macrophages^75^. In adipose tissue, senescent cells secrete inflammatory factors such as MCP-1 and MIP-1β, which attract macrophages into adipose tissue to participate in the inflammatory response, thereby promoting macrophage infiltration and accumulation. These macrophages further exacerbate inflammation and insulin resistance. Reducing senescent cells in obese mice was found to reduce plasma concentrations of the macrophage-attracting chemokines MCP-1, MIP-1β, and M-CSF^76^. In a model of nephrotoxic serum nephritis (NTN) in aged mice, macrophage infiltration in the glomeruli was significantly reduced, suggesting that aging impairs the ability of macrophages to infiltrate. In addition, the expression of cytokines and chemokine receptors that mediate macrophage infiltration, such as CCR1 and CCR2, were downregulated on kidney infiltrating macrophages, potentially affecting macrophage migration and immune function^77^.

##### 2.2.1.2 SASP is tightly linked to macrophage polarization

In the liver, macrophages tend to polarize toward the M1 phenotype with age. Studies have shown that aged macrophages have decreased levels of CD206 (an M2 marker) and increased levels of iNOS (an M1 marker). Interestingly, polarization can be influenced by macrophage autophagy, which is typically inhibited by ATG5. Compared to young mice, macrophages in aged mice have significantly fewer autophagosomes and LC3 puncta, and exhibit higher levels of pro-inflammatory cytokines that promote M1 polarization, such as TNF-α, IL-1β, and IL-6. Enhancing ATG5 expression to restore autophagy can modulate the polarization of aged macrophages, reducing the pro-inflammatory properties of the M1 phenotype and increasing the anti-inflammatory properties of the M2 phenotype^78^.

In the context of tumor cell senescence (TIS), these cells alter their secretory profile to form the so-called SASP, which includes components such as IL-6, IL-10 and TGF-β. These components can induce macrophage polarization from a pro-inflammatory M1 type to a pro-repair M2 type, which affects their phagocytic ability towards tumor cells^71^. Studies have shown that senescent cells, whether induced by radiation or chemical means in endothelial and preadipocyte cells in adipose tissue, secrete a series of inflammatory factors (such as IL-6, TNF-α, and IL-10) known as SASP. This secretion increases the number of M1-type macrophages (pro-inflammatory phenotype), which express high levels of CD38 and decrease NAD+ levels, thereby exacerbating senescence and inflammation^79^ In a mouse liver cancer study, SASP factors were found to further activate macrophages by increasing the expression of the total macrophage marker CD68. Over time, there is a gradual increase in the M2 macrophage marker CD163, while the M1 macrophage marker iNOS peaks early during induction and then declines^80^.

Kidney disease is considered a form of renal aging^77^. In a study of severe renal injury, young and aged C57BL/6 mice were subjected to bilateral renal ischemia-reperfusion injury (IRI) to examine renal function. Impaired M2 polarization was likely associated with reduced expression of macrophage CSF-1 and downregulation of interferon regulatory factor 4 (IRF4) signaling by arresting more proximal tubular cells in the G1 phase. *In vitro* experiments showed that bone marrow-derived monocytes from aged and young mice showed no difference in M2 polarization capacity under cytokine stimulation, suggesting that aging directly affects the renal microenvironment rather than the intrinsic polarization capacity of the macrophages themselves^81^.

In the skin, IL-33, a major component of the SASP secreted by senescent fibroblasts, is crucial for the repair of radiation-induced skin injury (RISI). Re-epithelialization and collagen deposition play critical roles in the recovery from RISI. IL-33 promotes re-epithelialization and collagen deposition in RISI by partially regulating the polarization of macrophages to the M2 type, thereby enhancing fibroblast activation, angiogenesis, and cell proliferation^82^.

A study of ocular neovascularization showed that macrophages in aged mice have an impaired function in inhibiting angiogenesis. This impairment is associated with a reduction in FasL on the surface of macrophages, resulting in a loss of their anti-angiogenic phenotype. IL-10 plays a critical role in the regulation of macrophage polarization and angiogenesis. The study found that the ocular microenvironment of aged mice has higher levels of IL-10, which may cause infiltrating macrophages to polarize to a pro-angiogenic M2 phenotype. In addition, macrophages in aged mice upregulate IL-10 expression after injury compared to young mice, but the expression of anti-angiogenic factors such as IL-12 and TNF-α is reduced^83^.

The above studies demonstrate the influence of senescent cells on macrophage polarization and the subsequent regulation of various tissue disease processes. However, not all SASPs are directly related to macrophage polarization. KMSCs recruit monocyte-derived Ly6C+CCR2+ macrophages that express pro-inflammatory cytokines through a number of senescence-associated features, but they do not significantly alter the expression of markers associated with the M2 phenotype^73^.

##### 2.2.1.3 SASP affects inflammatory signaling pathways and cytokines in macrophages

In a pulmonary study in mice, elevated levels of A20 were found to inhibit polyubiquitination of TRAF6 in macrophages, thereby attenuating activation of the NF-κB and MAPK pathways, leading to a reduced capacity of macrophages to produce inflammatory cytokines. Supplementation with fish oil lowered A20 levels, which could restore the response of aged macrophages to bacteria^84^. In a mouse hepatocellular carcinoma (HCC) experiment, the expression of Bcl3, an important factor in the NF-κB pathway in senescent hepatocytes, was increased. Experimental evidence showed that Bcl3 not only promotes the formation of the SASP phenotype, but also activates inflammatory signaling pathways in macrophages^80^. In breast cancer, Plk1-induced chronic chromosomal instability (CIN) led to cellular senescence, where the secreted SASP induced the influx of inflammatory monocytes and activated the NF-κB signaling pathway in macrophages, along with the expression of downstream inflammatory response genes^85^. In a study on colorectal cancer, senescence induced by the METTL3/CDKN2B axis could promote tumor progression by inducing M2 polarization of macrophages through SASP, leading to the expression of an anti-inflammatory phenotype^86^.

#### 2.2.2 Extracellular vesicle pathway and macrophages in senescent cells

EVs are specialized structures released by cells into the environment and include exosomes and microvesicles. They are recognized as key players in intercellular communication, closely associated with the immune system, and capable of exchanging lipids and proteins^87^. However, the extracellular vesicles secreted by senescent cells, as opposed to young cells, show increased numbers, altered characteristics, and modified transmission mechanisms due to factors such as DNA damage, lipid oxidation, and changes in their contents. These factors lead to significant differences in their effects on macrophages^88^. Here, we summarize the effects of senescent cell-derived extracellular vesicles on macrophages.

##### 2.2.2.1 Extracellular vesicles of senescent cells can affect the expression of macrophage cytokine and inflammatory signaling pathways

In the liver, THP-1 macrophages treated with EVs from senescent cells showed a significant increase in the secretion of EGF. This suggests that senescent cells directly influence macrophage function through their released EVs, particularly in regulating cell secretion^89^. Another study on the effects of senescent EVs on macrophages found that the expression levels of certain microRNAs (such as miR-146a, miR-21, miR-223, and let-7a) were significantly increased in the plasma EVs of aged mice. These microRNAs may affect macrophage function by regulating key signaling pathways within macrophages. Macrophages pretreated with senescent cell EVs expressed higher levels of Arg1, IL-10, MRC1, and TGF-β1, and reduced levels of IL-6 and iNOS after LPS stimulation, suggesting that senescent cell EVs tend to promote a more immunomodulatory phenotype in macrophages and reduce inflammatory responses^90^. In aged mice, the concentration of miR-192 in serum EVs was significantly increased, and it was further discovered that miR-192 regulates macrophage function by reducing the expression of inflammatory cytokines such as IL-6 and CCL2. In addition, miR-192 reduces the expression of these cytokines by targeting key molecules involved in inflammatory signaling within macrophages, such as NF-κB and TBK1, thereby alleviating inflammation in aged mice^91^. In addition, studies have shown that the number of EVs released from the liver of senescent mice is significantly increased compared to normal cells, and these vesicles are enriched for miR-30b-5p. These senescent cell EVs can be phagocytosed by surrounding macrophages, and the miR-30b-5p they carry can directly target the mRNA of SIRT1, reducing its expression, activating the NF-κB signaling pathway, and promoting the production and release of inflammatory cytokines by macrophages, such as IL-1β and IL-6, thus activating and enhancing the inflammatory response^92^. This suggests that senescent cell EVs may have both anti-inflammatory and pro-inflammatory effects. This variability may be related to the different sources of EVs used in the experiments, with anti-inflammatory EVs derived from cultured cells and others from plasma. These data demonstrate that the different origins of EVs and their contents may lead to different effects, which warrants further research.

##### 2.2.2.2 Senescent extracellular vesicles can have effects on macrophage activation, recruitment, polarization and phagocytosis

Hydrogen peroxide-induced senescent cholangiocytes (NHC-sen) or cholangiocyte-like structures from patients with primary sclerosing cholangitis (PSC) attracted more macrophages. In addition to an increase in SASP, NHC-sen released more exosomes. These exosomes, which contain FN, Alix, TfR, and CD81, may serve as important mediators for communication between cholangiocytes and macrophages, promoting macrophage recruitment and activation^93^. In experiments investigating the differential effects of aging and young mesenchymal stem cell (MSC)-derived EVs on acute lung injury (ALI), aging MSC-EVs showed higher levels of miRNAs that promote M1 macrophage polarization (such as miR-127-3p and miR-125b-5p) and lower expression of miRNAs that promote M2 polarization (such as miR-223-5p). This partially explains why aging MSC-EVs are less effective in promoting M2 macrophage polarization, thereby reducing their efficacy in anti-inflammatory and acute lung injury repair^94^. In addition, EVs from senescent cells can significantly increase the phagocytic activity of macrophages, and CD63+ depletion experiments indicate that specific EV subpopulations play a critical role in regulating macrophage function during senescence^90^.

#### 2.2.3 Senescent cell-mediated inhibition of phagocytosis of macrophages

**Figure 3:**
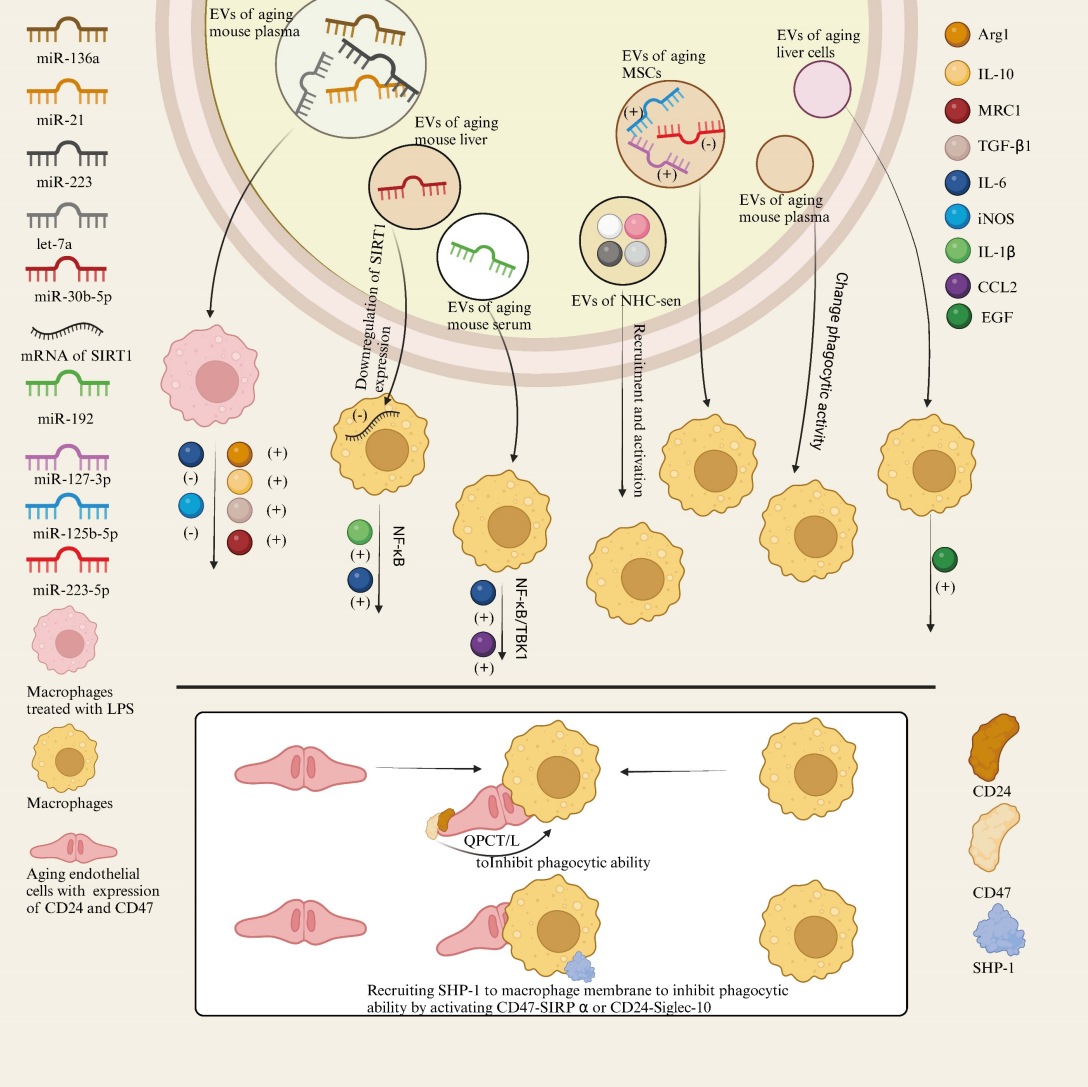
Mechanism of SCES and EVs-related effects of senescent cells and tissues on macrophages.

Suppression of SCES is independent of the SASP and instead requires direct contact between macrophages and senescent cells. SCES involves the inhibition of macrophage phagocytic capacity by senescent cells through increased expression of CD47, a process dependent on the activity of QPCT/L. In addition to CD47, increased expression of CD24 in senescent epithelial cells has been identified as critical for SCES. Specifically, in the absence of CD47, increased CD24 expression suggests that CD24 can partially compensate for the absence of CD47, further inhibiting the phagocytic ability of macrophages. Another key mechanism of SCES is the activation of SHP-1, a phosphatase recruited under the macrophage membrane via activation of the CD47-SIRPα or CD24-Siglec-10 axis, which suppresses macrophage phagocytic function^17^. The mechanism of SCES and EVs-related effects of senescent cells and tissues on macrophages is shown in Figure 3.

### 2.3 Characterization of senescence-associated macrophages

Senescent cells are continuously produced throughout life and play important roles in embryogenesis, wound healing, host immunity, and tumor suppression. However, with increasing age, the accumulation of senescent cells leads to niche occupancy and the production of pro-inflammatory cytokines, contributing to the development of age-related diseases^95^. Macrophages, as key phagocytes, experience functional impairment with age, leading to the accumulation of abnormal cells^96^, this results in compromised mechanisms for clearance of senescent cells and dysregulated immune responses mediated by the macrophages themselves. While senescent cells have been extensively studied in the past, the investigation of senescence-associated macrophages remains limited. For senescent cells, one study proposed twelve hallmarks of aging: genomic instability, telomere attrition, epigenetic alterations, loss of proteostasis, autophagy dysfunction, deregulated nutrient sensing, mitochondrial dysfunction, cellular senescence, stem cell exhaustion, altered intercellular communication, chronic inflammation, and niche dysregulation^68^. Given the critical role of senescence-associated macrophages in the body, we will discuss these features based on the existing literature. We will summarize the differences between senescence-associated macrophages and normal macrophages and the role of senescent macrophages in pathophysiology, focusing on the two broad features of metabolism and autophagy, and considering the unique functions of macrophage phagocytosis, infiltration, and polarization.

#### 2.3.1 Decreased autophagy levels and altered polarization states

Autophagy is one of the essential processes in eukaryotes that maintains cellular homeostasis by removing misfolded proteins, damaged organelles, and pathogens, and it also plays a role in cellular senescence^97^, ^98^. In experiments with Atg7 knockout mice, a gene essential for autophagy, it was found that their autophagy levels were similar to the decreased autophagy observed in aged mice. In addition, inhibition of ATG5 in senescent mice resulted in a significant decrease in autophagy levels. Even after stimulation with LPS and IFNγ, the number of autophagosomes in senescent macrophages was lower than in young macrophages^78^, ^99^, ^100^. Interestingly, overexpression of ATG5 restored autophagy in macrophages to levels comparable to normal macrophages and reduced the senescent phenotype^78^. This indicates that the level of autophagy in senescent macrophages is lower than that in normal macrophages. Some studies have shown that activation of autophagy in bone marrow-derived senescent macrophages can delay aging and regulate inflammation^101^. In tumor-associated macrophages, the expression of autophagy-related proteins LC3 and Beclin-1 can inhibit the expression of cyclin-dependent proteins p16 and p21, thereby delaying senescence^102^. In addition, some exosomes can affect the state of autophagy^103^. Overall, the level of autophagy in senescent macrophages is reduced but reversible, and when the level of autophagy is increased, the senescent state of macrophages can be reversed under certain conditions and improve the homeostatic capacity, although research in this area is still limited.

Notably, one study indicated that inhibition of autophagy not only promotes macrophage senescence and increases SASP levels, but also alters the composition of various factors within SASP^104^. Therefore, when reversing the impaired autophagic function in senescent macrophages, attention should be paid to the differential expression of factors induced by this reversal process compared to normal macrophages, as well as to the interactions between different cells.

Macrophage phenotypes are dynamic, even when a macrophage adopts a particular phenotype, it retains the ability to change under the influence of various factors in a new environment^97^. One study showed that bone marrow-derived macrophages from autophagy-impaired Atg5 knockout mice exhibited increased pro-inflammatory M1 polarization and decreased anti-inflammatory M2 polarization. Impaired autophagy in macrophages can promote M1 polarization and lead to chronic inflammation and damage in the liver of obese mice^105^. Sialic acid can inhibit the LKB1-AMPK-Sirt3 pathway, upregulate intracellular ROS, and impair the autophagolysosomal system, thereby blocking autophagic flux and leading to M1 polarization of RAW264.7 macrophages^106^. Mitochondrial ROS can cause lysosomal dysfunction and induce M1 polarization of macrophages under diabetic conditions by impairing autophagy^107^. Overall, inhibited or impaired autophagy often leads to M1 polarization of macrophages, whereas enhanced autophagy tends to promote M2 polarization. Since inhibition or impairment of autophagy also leads to senescence, senescent macrophages may undergo phenotypic changes compared to normal macrophages.

#### 2.3.2 Metabolic changes

Previous studies have found that the metabolism of macrophages in M1 macrophages primarily involves glycolysis, mainly regulated by HIF-1, while M2 macrophages have a higher baseline mitochondrial oxygen consumption rate than M1 macrophages. Therefore, M2 macrophages primarily rely on fatty acid oxidation, regulated by the PPAR family, and mitochondrial respiration^108^. Thus, when discussing the metabolism of senescent macrophages, these three metabolic pathways and the associated regulatory factors should be considered.

Studies have shown that NAD+ levels decrease in senescent cells^109^, and in senescent macrophages, the enzyme quinolinate phosphoribosyltransferase (QPRT) is inhibited, leading to reduced NAD+ synthesis. This results in decreased mitochondrial respiration and increased glycolysis^110^. An experiment investigating the effects of oxidative stress on macrophages from mice of different ages found that aged mouse macrophages exhibited lower levels of catalase (CAT) and glutathione (GSH), higher levels of ROS, and increased lipid accumulation^111^. Kupffer cells in the livers of aged mice also showed reduced respiratory burst activity, decreased phagocytic capacity, and increased oxidative stress compared to young mice^112^. Furthermore, NAD+ is also a necessary coenzyme for poly (ADP-ribose) polymerase (PARP), and insufficient NAD+ levels inhibit PARP activity, reducing DNA repair capacity, increasing genomic instability, and potentially leading to diseases such as cancer^113^, ^114^. Additionally, a decline in NAD+ levels can lead to increased oxidative stress and impaired mitochondrial function^115^. Notably, these NAD+-induced changes have also been observed in senescent macrophages^100^, ^116^-^119^.

Prostaglandin E2 (PGE2) is an important lipid signaling molecule that plays a role in lipid metabolism. Previous studies have shown that PGE2 levels are increased in senescent macrophages under LPS stimulation due to the upregulation of COX-2^120^, moreover, IL-1β promotes the production of PGE2 in senescent macrophages from aged mice, which is further supported by the finding that macrophages from aged mice fed with vitamin E produce more PGE2^100^, ^121^, ^122^. Research suggests that PGE2 can activate the cAMP-PKA pathway, leading to malfunction of the malate-aspartate shuttle (MAS) system and reducing ATP production. It also inhibits the oxidative metabolism of IL-4-induced M2 macrophages^123^ and affects macrophage polarization, which may be more pronounced in senescent macrophages. However, a study of resident alveolar macrophages (AMs) showed that although AMs in aged mice had increased phagocytic capacity, their ability to synthesize prostaglandins did not change^124^. This suggests that PGE2 levels in senescent macrophages may exhibit significant heterogeneity in different tissues, although research is currently lacking. One study found that TNF-α and PGI2 produced by rat macrophages increased with age^125^. While studies have shown that levels of thromboxane B2 and arachidonic acid decrease with increasing metabolic capacity, these results are limited to AM in neonatal (10-day-old) and adult (2-month-old and 4-month-old) mice^126^, changes in these compounds in senescent macrophages or aged individuals have not been documented. Based on the tendency of senescent macrophages to polarize toward the M1 phenotype, we can speculate about changes in arachidonic acid and thromboxanes, but further research is needed.

Overall, senescent macrophages tend to polarize towards the M1 phenotype and exhibit glycolytic metabolism. This is associated with decreased mitochondrial function, increased oxidative stress, chronic inflammation, reduced activity of various enzymes, decreased respiratory substrates, and a range of downstream mechanistic changes. From a lipid metabolism perspective, PGE2 levels in senescent macrophages are heterogeneous but show an increasing trend, influencing macrophage polarization. However, research on other prostaglandins and their precursors remains limited.

#### 2.3.3 Changes in gene regulatory function

Blacher E found that the phagocytic activity of aged macrophages and the diurnal rhythm of monocyte migration from bone marrow to blood in aged mice were disrupted. This is due to the breakdown of circadian genes in senescent macrophages, with only 58 genes remaining active compared to 680 in normal macrophages. Further investigation revealed that the Klf4 gene plays a crucial role in regulating circadian rhythms, controlling phagocytic activity and the expression of related genes^127^. In a study on AMs, it was found that gene expression in senescent AMs changed, with downregulation primarily affecting cell cycle-related pathways, such as genes involved in the mid-cell cycle checkpoint like CCNB1 and BUB1, and mitotic initiation genes like AURKA and CDK1^128^. Transcriptome analysis of cardiac-resident macrophages showed that genes related to the complement cascade, pro-inflammatory responses, and RNA splicing enzymes were upregulated in senescent macrophages. During aging, there was an increase in CCR2+ and a decrease in CCR2- cardiac-resident macrophages, accompanied by downregulation of wound healing-related genes^129^. In a study on glomerulosclerosis in aging females, macrophages exhibited increased expression of pro-inflammatory genes. RT-PCR analysis showed that the mRNA levels of RANTES, VCAM-1, and MMP-12 increased by 5.3-fold, 3.5-fold, and 3.3-fold, respectively^130^.

On the whole, research on gene regulation in senescence-associated macrophages is limited. Existing studies have primarily focused on circadian rhythm genes, genes related to phagocytic activity, cell cycle-related genes, and genes associated with pro- and anti-inflammatory tissue repair. Similar to other senescent cells, senescence-associated macrophages exhibit circadian dysregulation, reduced phagocytic capacity, cell cycle arrest, and a tendency toward pro-inflammatory gene expression. Alterations in gene regulatory functions in these macrophages remain an area for further investigation.

#### 2.3.4 Reduced phagocytic ability

Macrophages have the ability to phagocytose cellular debris and foreign bodies, as well as digest pathogens. Studies have shown that senescent macrophages exhibit reduced phagocytic activity due to circadian rhythm disruptions that affect gene expression^127^. Senescent alveolar macrophages (AMs) have decreased ability to phagocytose apoptotic neutrophils and internalize fluorescent particles, along with significantly reduced expression of CD204 (scavenger receptor-A)^128^. In another study of the LC3-associated phagocytosis (LAP) pathway in macrophages during Streptococcus pneumoniae infection, macrophages from aged mice (20 to 22 months old) not only lacked LAP and bacterial killing ability compared to those from young mice (2 months old), but also produced higher levels of pro-inflammatory cytokines^131^. MYC is an oncogenic transcription factor, and USF1 is a transcription factor that regulates the expression of metabolism-related genes. The activity of MYC and USF1 is reduced in senescent macrophages, leading to a decrease in phagocytic capacity similar to that observed in MYC and USF1 knockout monocyte-derived macrophages^132^. The Axl receptor, one of the key receptors for phagocytosis of apoptotic cells, is significantly reduced in aged bone marrow macrophages, directly contributing to the decline in their phagocytic function^133^. P53 is a tumor suppressor gene, and studies have found that macrophage phagocytic activity in aged mice can be inhibited by P53. In addition, P53 can affect c-Myc, thereby reducing the expression of M2-associated genes^134^.

The intracellular space of macrophages can influence their phagocytic capacity. During macrophage metabolism, increased oxidative stress, decreased antioxidant capacity, and the accumulation of lipofuscin can encroach upon the normal cellular space, thereby impairing phagocytic function^111^. A similar situation may also occur in testicular cells of aged mice, where increased lipid accumulation has been observed^135^.

Impaired chemotactic ability may also lead to reduced phagocytic capacity. Studies have shown that in senescent AMs, cell surface expression and redistribution of the scavenger receptor MARCO is significantly reduced. In addition, Rac1 mRNA and protein expression levels are decreased, resulting in reduced Rac1 GTP levels. This in turn affects the activation of the Arp2/3 complex and F-actin polymerization, resulting in fewer filopodia, which negatively affects phagocytic capacity^136^.

In general, senescence-associated macrophages exhibit a decrease in phagocytic capacity. Contributing factors include negative regulation by genes and cytokines, encroachment on cellular space, and a reduction in chemotactic ability.

#### 2.3.5 Changes in migration and recruiting capacity

The migration, recruitment, and chemotactic abilities of macrophages are fundamental to their cellular functions, and aging can affect these functions. A recent study found that silencing MYC and USF1 in bone marrow-derived macrophages results in reduced chemotactic activity. In senescent macrophages, the activity of these two genes is lower, suggesting that the chemotactic ability of senescent macrophages may be altered due to changes in the activity of MYC and USF1^132^.

In a nephrotoxic serum (NTS) nephritis mouse model, the infiltration of F4/80-positive macrophages in the glomeruli of aged mice was significantly reduced, possibly related to CCL1 and CCL2^77^. A study on glomerulosclerosis suggested that TNF-α may increase macrophage infiltration in aged mice by promoting pro-inflammatory mesangial cells^130^. In addition, research has shown that follicle-stimulating hormone in aged females can lead to increased secretion of IL-8 by renal proximal tubular epithelial cells, which enhances macrophage infiltration and pro-inflammatory activity, exacerbating tubular fibrosis^137^.

#### 2.3.6 Changes in antigen-presenting capacity

Macrophages are the primary antigen-presenting cells in the body. In addition to presenting antigens to CD4+ T cells via MHC class II molecules for pathogen recognition, they also use pattern recognition receptors (PRRs) on their surface and secrete costimulatory molecules and chemokines to control various physiological activities. One study found that elevated levels of A20 in the lungs and alveolar macrophages of aged mice inhibit the ubiquitination of the signaling protein TRAF6, thereby suppressing the NF-κB and MAPK pathways, which affects the PRR-based antigen-presenting ability of macrophages^84^. In addition, there is a certain degree of competition between the phagocytic function and the antigen-presenting function related to the inflammatory response. Research has shown that in senescent bone marrow macrophages, the inflammatory signals CD86 and MHC class II are increased, leading to a greater propensity for inflammation rather than phagocytosis at this stage^133^. Furthermore, senescent macrophages exhibit decreased mitochondrial calcium uptake, which triggers inflammation, a process associated with the Mcu gene^138^.

In the kidneys of aged mice, the expression of Fcγ receptors on macrophages is significantly reduced, which may lead to a decrease in inflammatory responses and reduced kidney damage, although serum levels of TNF-α and IL-6 are increased^77^.

#### 2.3.7 Changes in the ability to secrete exosomes

A study found that senescent RAW 264.7 mouse macrophages exhibit increased secretion of EVs, which contain various pro-inflammatory miRNAs and mRNAs, with most of the proteins involved in positively regulating EV secretion. The contents of these EVs and the activation of the NF-κB pathway may promote the proliferation of mouse embryonic fibroblasts and inhibit the early senescence of tumor cells, potentially facilitating tumor development^139^. Similarly, senescent macrophages induced by high glucose also show an increase in exosomal membrane proteins^140^.

### 2.4 Effects of senescent macrophages on other cells

Existing research suggests that senescent macrophages affect other cells primarily through the secretion of the SASP, thereby promoting inflammation and fibrosis. Studies have shown that senescent macrophages can release factors such as IL-6 and TNF-α via the SASP pathway, affecting renal cells and influencing the inflammatory state^141^. Under hyperglycemic conditions, senescent macrophages highly express glucose transporter 1(GLUT1) and secrete factors such as IL-1β, promoting inflammation in periodontal tissue cells and contributing to osteoporosis. In addition, SASP can enter the bloodstream, induce systemic chronic inflammation, and exacerbate diabetic complications^141^. In addition, senescent macrophages under hyperglycemic conditions also play a role in promoting fibrosis. One study found that macrophages in diabetic mice exhibit senescent tendencies and produce a CXCR2 ligand-enriched SASP that acts on fibroblasts to promote fibrosis^142^. In addition, radiation-induced senescent macrophages can secrete TGF-β1 and Arg-1, which stimulate the fibrotic phenotype of lung fibroblasts and promote the development of lung fibrosis^143^. In mdx/utrophin-deficient mice (a severe model of Duchenne muscular dystrophy), macrophages also exhibit senescent characteristics, inhibiting the proliferative and regenerative functions of muscle stem cells through SASP and promoting fibrosis^144^.

SASP may also promote tumor cell metastasis and immune evasion. In a KRAS-driven lung cancer model, the SASP secreted by senescent macrophages includes chemokines such as CCL2, CCL8, CCL7, CCL24, and CXCL13, which are involved in tumor cell metastasis. In addition, factors such as CCL7 and CXCL13 can recruit regulatory T cells and reduce effector T cells, thereby reducing immune surveillance^145^. SASP may also promote angiogenesis and senescence. Studies have found that senescent macrophages promote angiogenesis during endplate sclerosis by secreting IL-10^146^. In KRAS-driven lung cancer, IL-10 and BMP2 in the SASP can act on vascular endothelial cells to promote angiogenesis^145^. Senescent macrophages can also release IFITM3, which acts on vascular smooth muscle cells, leading to vascular calcification and senescence^147^. Interestingly, SASP secreted by senescent macrophages can also affect normal macrophages. Senescent macrophages induced by SARS-CoV-2 can activate surrounding macrophages into a pro-inflammatory phenotype and recruit more macrophages by secreting IL-1α, IL-6, IL-8, and TNF-α^148^.

Additionally, research has shown that senescent macrophages can influence other cells through EVs. The EVs secreted by senescent macrophages can act on mouse embryonic fibroblasts, containing components such as miR-21a or Mvp, and promote their proliferation through the activation of the NF-κB pathway^139^.

### 2.5 Markers of senescence-associated macrophages

Biomarkers at the cellular level refer to molecules that can be detected and measured within cells, typically reflecting the functional state, disease condition, or exposure to specific environments. These biomarkers can include proteins, nucleic acids (DNA or RNA), metabolites, or specific molecules on the cell surface. In the study of senescent cells, commonly used markers of senescence include SA-β-gal, p16^Ink4a, p21, and SASP.

#### 2.5.1 Common senescence markers in macrophage senescence

SA-β-gal is a lysosome-derived β-galactosidase enzyme^149^ whose activity increases during cellular senescence, making it a commonly used marker for the detection of senescent cells. In experiments, SA-β-gal has been used as a probe to detect senescent cells such as lung epithelial cells^150^, retinal pigment epithelial cells^151^, and hepatic stellate cells^152^. In addition, it has been developed as a prodrug in senescence-related studies. For example, CHANG *et al.* used SA-β-gal as a prodrug to investigate the role of SA-β-gal-responsive PROTAC molecules in inducing apoptosis of senescent cells in the tumor microenvironment^153^. However, because SA-β-gal can also exhibit increased activity in non-senescent cells, it may lead to false-positive results in the identification of senescent cells, such as in certain retinal neurons^154^ and macrophages^155^. Therefore, it is not suitable as a sole biomarker to identify senescent macrophages.

P21 can independently induce the senescence program^156^. However, when DNA damage or other stressors occur, activation of the tumor suppressor p53 leads to transient expression of p21, which can induce temporary G1 cell cycle arrest or stabilize chromosomes in association with apoptosis^157^-^159^. In addition, research has shown that its overexpression is a key feature of liver inflammation and carcinogenesis caused by loss of the NF-κB pathway regulator NEMO^160^. Although p21 can influence macrophage polarization, reprogramming, and chemotaxis^161^, ^162^, it cannot yet be considered a definitive marker of their senescent phenotype.

p16, also known as CDKN2A, is a tumor suppressor protein encoded by the CDKN2A gene. In past studies, p16 was often used as a marker of cellular senescence. However, some research has indicated that its expression in macrophages is inconsistent. For instance, p16 can be induced by immune stimulation rather than by cellular senescence, as macrophages lose this marker under M1-inducing stimuli but increase its expression under M2-inducing stimuli, without undergoing senescence. This phenomenon similarly applies to SAβG^163^. Additionally, studies have found that p16 expression lacks specificity, as age-induced p16 accumulation is a slow process and occurs in vascular endothelial cells, macrophages, and adipocytes^164^.

In addition, markers such as γ-H2AX, which increases during cellular senescence and DNA damage response, along with various nuclear features, protein dysregulation, and telomere shortening, represent a wide range of universal features of senescence. We will not delve into these specifics in this discussion.

#### 2.5.2 Development and Screening of Markers for Macrophage senescence

For specific cells such as macrophages, many experiments identify senescence by changes in multiple markers. For example, in a study investigating the effect of senescence on muscle atrophy in chronic kidney disease, researchers used a combination of SA-β-gal, the DNA damage response marker γ-H2AX, and the senescence pathway markers p21, p16, and p53 to identify senescence^165^. In addition, to more accurately identify senescent macrophages, it is important to study their specific markers. Research has shown that lymphatic vessel endothelial hyaluronan receptor 1 (LYVE1), granulin (GCA), CD22, GLUT1, CD38, CD9, CD206, TREM2 and Gpnmb can serve as specific markers to identify senescent macrophages[100, 166, 167][168][169, 170].

Lymphatic vascular endothelial LYVE1 is a member of the Link protein superfamily containing a conserved HA-binding domain (Link module). Flow cytometric analysis and clustering of macrophages revealed that in aged male mouse skeletal muscle, LYVE1- macrophages are more abundant than LYVE1+ macrophages^171^. In addition, research has found that the proportion of LYVE1+MHCII^lo macrophages in the hind paws of diabetic mice significantly increased between 12 and 21 weeks^172^.

Although research on senescent macrophages is limited, it is evident that changes in LYVE1 often occur in parallel with changes in MHC II. In fact, previous studies have reported that in aged macrophages, the expression of MHC class II genes, particularly the IA complex on the cell surface, as well as the levels of intracellular IAβ protein and mRNA, are reduced. Transcription of the IAβ gene is impaired in aged macrophages^173^, ^174^. In addition, research has shown that the expression of PRMT5 in macrophages is downregulated in senescent and H2O2-treated macrophages, leading to ineffective induction of MHC II transcription by IFN-γ, thereby reducing MHC II expression^175^.

GCA is a calcium-binding protein. Researchers have discovered that senescent macrophages in calluses can induce senescence in skeletal stem/progenitor cells (SSPCs) by secreting GCA^176^. In addition, genetic deletion of GCA in macrophages can delay skeletal aging^177^.

CD22 is a leukocyte differentiation antigen that binds to its ligand CD47 and plays a role in immune regulation. Microglia are the resident macrophages of the brain, and researchers have found that CD22, identified as a regulator of microglial phagocytosis, is more highly expressed in aged microglia compared to adult microglia^167^, ^178^. CD38 is a transmembrane surface protein that is often overexpressed in tumor cells. In a study of tumor-induced senescent macrophages, senescent macrophages were found to express high levels of CD38 along with increased expression of Arg1, which is associated with decreased T cell reactivity^166^.

CD9 is a transmembrane protein involved in activities such as signal transduction. In a study on the effects of obesity-induced senescent macrophages on adipocyte progenitor cells, it was found that this macrophage subset highly expresses CD9 and exhibits senescent characteristics, promoting the fibrosis process^168^. Additionally, studies have found that CD9 can promote senescence in various cells, including macrophages, through the PI3K-AKT-mTOR-p53 signaling pathway. Interestingly, research has also shown that CD206 expression decreases in senescent macrophages, which may be related to changes in polarization state. This finding warrants further investigation.^169^

TREM2 is a transmembrane receptor expressed on the surface of myeloid cells. In a study on Alzheimer's disease, TREM2-deficient mice exhibited fewer microglia with senescent characteristics, suggesting that TREM2 is a potential marker of senescent macrophages^170^.

Gpnmb is a transmembrane glycoprotein that is often upregulated in aging macrophages. Studies have found that its expression significantly increases in macrophages within aged skeletal muscle. In single-cell RNA sequencing analysis of skeletal muscle, Gpnmb is highly expressed in specific macrophage clusters (such as cluster Cl5) in aged mice. These clusters also show upregulation of other genes related to fatty acid transport and inflammation, such as Fabp4 and Fabp5^171^.

GLUT1 is a glucose transporter that facilitates the passage of glucose across the cell membrane. Single-cell sequencing has shown that senescent macrophages have an impaired glycolytic transcriptome associated with inhibition of GLUT1^179^. Markers are shown in Table 1.

## 3. Macrophage in kidney

### 3.1 The role of macrophage in renal development

The concept of nutritive macrophages has gradually led us to recognize the series of changes macrophages induce in angiogenesis, lymphatic development, organogenesis, neural network formation, myogenesis, and adipogenesis^180^. The kidney, being rich in vasculature, lymphatics, and ducts, relies significantly on macrophages during its development. Given the involvement of macrophages in pro-inflammatory and reparative processes, immune regulation, and tissue clearance processes also relevant to renal pathology, here, this discussion primarily focuses on the nutritive role of macrophages in kidney development.

#### 3.1.1 Kidney formation

Macrophages arrive in the kidney between E8.5 and E12, although the precise timing remains unclear^181^, ^182^. Earlier studies have shown that macrophage CSF-1 can increase macrophage numbers, thereby promoting kidney growth and differentiation. This may be related to the release of growth factors or direct contact, but it is evident that an increase in macrophage numbers accompanies the branching of the kidney and the formation of nephrons^181^. Subsequent studies have further confirmed that CSF-1 indeed promotes macrophage proliferation, which in turn enhances renal cell proliferation through IGF-1, manifesting in increased kidney weight and changes in macrophage phenotypes in neonatal mice^183^. Recent research has revealed that macrophages align along nephron progenitor cells and participate in the clearance of these progenitors and ureteric bud development, involving macrophage phagocytosis and the release of growth factors such as VEGF^182^.

#### 3.1.2 Lymphatic duct formation

At E13, renal lymphatic vessels, marked by lymphatic endothelial-specific hyaluronan receptor (LYVE1)+, connect with external lymphatic vessels. However, macrophages arrive in the kidney before this process occurs, prompting further consideration of their potential role in lymphatic duct formation^184^. Previous research has indicated that macrophages can release VEGF-C through a VEGFR3-dependent mechanism to promote lymphatic proliferation^185^. Recent studies reaffirm this, demonstrating that a subset of VEGFR4+ macrophages exhibits strong chemotactic activity towards VEGF-C^186^. Upon stimulation by VEGF-C, macrophages release CD137L, which binds to CD137 receptors on lymphatic endothelial cells (LECs), activating autophagy. This activation enhances the proliferation, migration, and tube formation capabilities of LECs, contributing to lymphangiogenesis. However, these findings are based on mechanisms observed in adult kidneys, and the exact mechanisms during embryonic development require further exploration^187^.

#### 3.1.3 Angiogenesis

In experiments involving antibody-mediated macrophage depletion, F4/80+CD206 macrophages were found to play a role in vascular development within the developing kidney, directly interacting with endothelial cells of small-caliber vessels, while aligning parallel to, but not in contact with, endothelial cells of larger vessels. These macrophages support vascular anastomosis and crosslinking through the secretion of VEGF factors, with their absence leading to discontinuous renal endothelial structures^182^.

Beyond the substantial impact of nutritive macrophages on the developing kidney, research has characterized a subset of Galectin-3 (Gal3) myeloid cells involved in immune responses within the kidney, coexisting with F4/80+CD206 macrophages. These macrophages may protect the developing kidney by clearing maternal antibodies and other substances that cross the placental barrier^182^.

### 3.2 The specific role of macrophage in AKI and CKD

Due to the limited regenerative capacity of the kidneys, acute kidney injury (AKI) often results in renal damage and functional decline, leading to the development of CKD. Clinically, CKD is commonly defined by the glomerular filtration rate and albuminuria^188^. However, factors such as age increase the inaccuracy of this definition, prompting extensive research in the field^189^. From the perspective of macrophages, the transition from AKI to CKD appears to be a continuous process. Herein, we briefly summarize the role of macrophages in the progression from AKI to CKD.

In the early stages of AKI, chemokines such as CC and CX3C and their corresponding receptor families play a direct role in recruiting macrophages^190^-^192^. Additionally, certain cytokines indirectly recruit macrophages by inducing the expression of chemokines. For example, the absence of IL-18 reduces the number of M1 macrophages on day 14 and M2 macrophages on day 30, indicating its influence on early M1 macrophage recruitment and pro-inflammatory factor release, as well as the repair function of later-stage M2 macrophages^193^. Moreover, pathogen-associated molecular patterns (PAMPs) and damage-associated molecular patterns (DAMPs), apoptosis-related proteins (e.g., GSDME), and oxidants (e.g., TMAO) are key factors in recruiting monocytes and activating resident macrophages^194^-^196^.

Once macrophages are recruited or activated, they predominantly exhibit an M1 phenotype in the early stages and actively promote inflammation through the release of inflammatory cytokines (such as TNF-α, IL1β, IL-6), oxidants and nitrogen species (e.g., Prdx1), upregulation of major histocompatibility complex II (MHC-II), and enhancement of inflammatory signaling pathways (such as NF-κB and MAPKs). These mechanisms and factors play a proactive role in promoting inflammation^104^, ^195^-^198^. However, previous studies have reported that anti-inflammatory repair and fibrosis mechanisms coexist within the early pro-inflammatory milieu. Besides the intrinsic negative regulatory molecules of macrophage inflammation (such as MCPIP1/Regnase-1)^199^, key inhibitory factors like AIM2 (induces macrophage pyroptosis)^200^ and neuropeptide Y (NPY) (inhibits M1 macrophage activation)^201^, as well as pro-fibrotic factors like MMP9 secreted by macrophages^198^, are involved in this process. This phenomenon may occur immediately after tissue injury as a feedback mechanism to prevent excessive inflammatory damage and promote subsequent tissue repair. Hence, it is not surprising that the transition of macrophages from a pro-inflammatory to an anti-inflammatory and reparative phenotype occurs post-injury, with macrophage depletion during this phase leading to delayed recovery, indicating the functional role of macrophages in renal repair^202^.

In the later stages of AKI, the reparative and anti-inflammatory actions of macrophages are largely carried out by M2 macrophages, which express markers such as CD206, CD36, and Arg1 (with CD163 being the primary phenotype in humans). The main mechanisms involve the secretion of anti-inflammatory cytokines (e.g., IL-10), growth factors (e.g., VEGF for vascular repair), phenotype conversion factors (e.g., IL-4), specific substances (e.g., SOD as an antioxidant enzyme), and the clearance of damaged tissues that continue to release pro-inflammatory signals. However, the excessive release of reparative factors like TGF-β may shift the repair process toward a pathological state, promoting the transition from AKI to CKD and advancing tissue fibrosis^203^-^207^.

In the early stages of CKD, similar mechanisms to those in AKI are present, involving chemokines, adhesion factors, DAMPs, and PAMPs, leading to the recruitment of macrophages. However, these signals are persistently expressed, continuously inducing macrophage migration and adhesion. The combined action of IFN-γ and TNF promotes the secretion of pro-inflammatory factors by macrophages, creating a self-perpetuating cycle of injury and inflammation. This mechanism underlies the chronic low-grade inflammatory state of the kidneys in CKD, where anti-inflammatory and reparative responses are relatively insufficient^196^.

During CKD repair, both M1 and M2 macrophages coexist and exert sustained effects. The prolonged release of cytokines by M2 macrophages and their transformation often result in renal tissue fibrosis. For example, macrophages can differentiate into myofibroblasts through the TGF-β1/Smad3 signaling pathway, JAK3/STAT6 signaling pathway, and downstream Src molecules^208^, regulating macrophage polarization, fibroblast activation, matrix metalloproteinase (MMP) and collagen release through the HIF-1/2α signaling pathway, and further exacerbating renal function decline by disrupting normal tissue structure through the interaction of Wnt/β-catenin and TGF-β pathways^209^.

## 4. The role of senescence-associated macrophages in renal diseases

### 4.1 The impact of senescent cells on macrophages in renal diseases

The glomerulus is a vital component of the kidney, responsible for filtering blood and regulating fluid balance. It is composed of podocytes, endothelial cells, mesangial cells, and other elements. The renal tubule, consisting of the proximal convoluted tubule, distal convoluted tubule, Henle's loop, and collecting duct, works in concert with the glomerulus to filter and maintain fluid equilibrium. In the connective tissues outside the glomeruli and tubules, renal interstitial cells are distributed, among which vascular endothelial cells are a primary focus in the study of kidney diseases. Numerous studies have reported that senescence of glomerular endothelial cells and podocytes in aged mice contributes to glomerulosclerosis or other deleterious effects on the glomerulus^210^, ^211^. Renal tubules are susceptible to hypoxia, toxins, metabolic disorders, and other factors, which drive the progression of kidney diseases, including acute kidney injury caused by damage and chronic kidney disease induced by aging^212^, ^213^. However, research on the role of senescent macrophages in the kidney remains limited. Here, we summarize the effects of senescent renal cells on macrophages to explore the mechanisms by which these cells promote kidney diseases.

#### 4.1.1 Glomerular cells

In a study on glomerulosclerosis in aged female mice, it was found that elevated TNF-α in the serum and increased NF-κB in senescent glomerular mesangial cells led to a shift towards a pro-inflammatory phenotype. This transition resulted in the upregulation of genes associated with macrophage infiltration, such as RANTES and VCAM-1, mediated by TNFR1, thereby increasing macrophage infiltration^130^. Various genes change expression in aged glomeruli, with NF-κB p50 protein primarily translocating to podocytes and parietal cells, leading to NF-κB release, which activates macrophage signaling pathways, contributing to inflammation and fibrosis. Additionally, the upregulation of VCAM and ICAM proteins in aged glomeruli stimulates macrophage adhesion and migration^214^.

#### 4.1.2 Tubular cells

SerpinB2, expressed in macrophages, inhibits the release of inflammatory factors. However, in senescent tubular cells, the increased expression of SerpinB2 elevates CCL2 levels, attracting macrophages to migrate for inflammation and repair, a mechanism particularly significant in chronic kidney disease^215^. VEGF, primarily expressed in glomerular podocytes and proximal tubule epithelial cells of the outer medulla, is notably reduced in the aged kidney's outer and inner medulla. This suggests that the decrease in VEGF in senescent proximal tubule epithelial cells correlates with reduced macrophage infiltration in the renal interstitium^216^. Senescent tubular cells exhibit G1 phase cell cycle arrest and secrete reduced levels of CSF-1, directly inhibiting macrophage polarization to the M2 phenotype through impaired CSF-1/IRF4 pathway activation, resulting in chronic inflammation and fibrosis. This mechanism explains the transition from acute kidney injury to chronic kidney disease^81^. In aged mice, increased LCK expression in tubular epithelial cells may lead to elevated serum leptin levels and enhanced phosphorylation of STAT3 and NF-κB in renal tissue, thereby increasing macrophage infiltration and tubular injury^217^. In type 1 diabetic mice, hyperglycemia induces senescence and iron accumulation in proximal tubular cells via the p21 pathway, indirectly promoting macrophage infiltration, which appears to be associated with p21^218^. Moreover, studies have demonstrated that AP-1 serves as a regulatory transcription factor in renal inflammation in mice, with its underlying mechanism involving the modulation of cellular senescence in renal tubular epithelial cells, thereby influencing the inflammatory response of macrophages^219^. Interestingly, macrophage-derived exosomes can induce tubular cell senescence, for instance, studies have demonstrated that miR-155 contained within exosomes derived from macrophages can be internalized by renal tubular cells, leading to telomere shortening and dysfunction via targeting TRF1, thus promoting renal fibrosis and tubular cell senescence^220^. These findings indicate that multiple factors within renal tubular cells can regulate cellular senescence, critically impacting macrophage function and contributing to the subsequent progression of renal disease. Consequently, targeting these factors offers novel prospects for the development of anti-aging and injury intervention strategies in nephrology.

#### 4.1.3 Renal endothelial cells

In the study of ischemia-reperfusion injury in aging kidneys, an increase in the expression of Neuropilin-1 (NRP1) and hypoxia-inducible factor 2a (HIF2a) was observed in endothelial cells (ECs). This upregulation recruits macrophages via the secretion of stromal cell-derived factor 1 (SDF1), which acts on the CXCR4 receptor. Concurrently, NRP1 and HIF2a suppress the expression of endothelial protein C receptor (EPCR), leading to platelet activation and the secretion of IL-1a. This cascade results in the overexpression of TIMP1 in macrophages, which, along with b1 integrin, activates fibroblasts and promotes tissue fibrosis^221^. Studies have shown that endothelial cells deficient in eNOS in aged mice trigger the release of von Willebrand factor (vWF), which deposits in renal arterioles. The local thrombosis caused by endothelial injury mechanisms induces localized inflammation (such as the generation of large amounts of reactive oxygen species, TNF-α, IL-1β, MCP-1) and attracts macrophages to participate in repair. The macrophages, in turn, release factors that stimulate collagen synthesis and fibroblast proliferation, leading to fibrosis. CD73+ stromal cells in the aging kidney secrete CCL2, inducing monocyte migration, differentiation into macrophages, and secretion of pro-inflammatory factors. In this scenario, macrophages further perpetuate the pro-inflammatory mechanism of CD73+ cells through feedback loops, ultimately leading to chronic kidney disease^73^.

#### 4.1.4 Research on various renal cells

In addition to the specific effects of senescent cells on macrophages, multiple types of senescent cells can simultaneously influence macrophages and mediate the onset of renal diseases. Studies on the changes in macrophages within the glomeruli of aged mice have found that the SASP released by senescent tubular epithelial cells, endothelial cells, mesangial cells, and lymphocytes leads to a decrease in chemokine receptors (CCR1, CCR2) and Fc gamma receptors (FcyⅠ-Ⅳ) on the surface of F4/80-positive, Ly-6G-negative macrophages, thereby affecting macrophage infiltration^77^. The reduced expression of H2S-generating enzymes (CBS and CSE) in aged kidneys leads to decreased H2S levels, while increased miR-21 expression further suppresses these enzymes. Together, these factors contribute to an increase in M1 macrophages and a decrease in M2 macrophages, resulting in a predisposition toward fibrosis and inflammation^222^.

### 4.2 The impact of aging-associated macrophages on kidney disease

Macrophages play a pivotal role within the body's immune system, and as the organism ages, their functions and characteristics undergo significant changes. In addition to the influence that aging tissue cells exert on immune cells, senescent macrophages themselves can induce functional alterations in tissue cells. In kidney diseases, these aging macrophages are instrumental in the progression and development of the condition. For instance, the reduced phagocytic capacity, diminished antigen-presenting function, abnormal secretion of inflammatory factors, and altered gene expression profiles of senescent macrophages are crucial in regulating the progression of kidney disease.

Recent research focusing on ferroptosis and aging macrophages has revealed that during the aging process of the kidney, macrophages exhibit signs of senescence alongside ferroptotic characteristics. Under the regulation of the ferroptosis-related Stat1/Pcbp1 signaling axis, these senescent macrophages experience iron homeostasis imbalance, thereby inducing oxidative stress mechanisms, such as increased lipid peroxidation and ROS accumulation, through ferroptotic signaling. In this context, senescent macrophages release SASP, where pro-inflammatory and pro-fibrotic molecules (TGF-β) and inflammatory cytokines further interact with tubular cells, promoting inflammation and epithelial-mesenchymal transition (EMT), leading to fibrosis^18^.

Additionally, an analysis has found that in kidney fibrosis diseases, during senescence, the proportion of SPP1+ macrophages polarizing into SPP1+MAM+ macrophage increases and is positively correlated with fibrosis, conserved across all fibrotic tissues, driven by regulators such as NFATC1 and HIVEP3. The gene expression of macrophages is upregulated, including matrix metalloproteinases (MMP7, MMP9, MMP19), tissue inhibitors (TIMP1, TIMP3), and endopeptidases (CTSB, CTSL, CTSH, CTSD, CTSZ), which interact with kidney cells and mediate extracellular matrix remodeling and fibrosis. This new macrophage polarization subtype plays a crucial role in the process of kidney fibrosis^223^.

In diabetic mice, hyperglycemia induces the expression of SASP in macrophages. Studies have found that in such conditions, senescent macrophages secrete increased levels of factors such as IL-1α and IL-6, promoting chronic inflammation in the kidney^224^. In line with studies on diabetic senescent macrophages, the inflammatory factors secreted by these cells may enter the bloodstream, triggering systemic inflammation. This mechanism can exacerbate diabetes and chronic nephritis, further worsening chronic kidney disease^141^. However, recent research has observed that senescent RAW264.7 mouse macrophages treated with hyperglycemia did not exhibit significant SASP protein expression; instead, they showed increased exosomal membrane protein expression. Thus, whether hyperglycemia truly induces increased SASP expression in senescent macrophages remains to be further studied^140^.

In LPS-induced macrophages, aging markers such as p16, p21, and β-galactosidase activity increase, and senescent macrophages release transmembrane protein 3 (IFITM3), promoting vascular smooth muscle cell (BSMC) calcification and vascular aging (with markers such as p16, p21 also increasing). These changes contribute to the onset of chronic kidney disease^147^.

Some studies suggest that senescent renal interstitial macrophages are associated with reduced VEGF levels. Their accumulation can downregulate the expression of VEGF in nearby renal tubules, leading to capillary rarefaction, resulting in tubular ischemia, cytokine release, and extracellular matrix deposition, thus exacerbating fibrosis. However, the studies only highlight senescence in the renal interstitium, without confirming whether the macrophages themselves are senescent. Therefore, further exploration of whether the accumulation of senescent macrophages leads to similar outcomes in the aging kidney repair process is particularly important^216^. In another study on glomerular senescence, macrophages were similarly affected. The results indicated that macrophages tended to express pro-inflammatory factors, which, although differing from the VEGF reduction mechanism caused by senescent renal interstitial macrophages, still favored a pro-inflammatory M1 macrophage phenotype, with reduced repair capacity^214^. This hypothesis is supported by evidence showing that during the transition from AKI to CKD, the number of M1 macrophages increases, tending towards long-term pro-inflammatory fibrosis induction^81^. Notably, the specific phenotype of aging-associated macrophages remains unclear.

**Figure 4:**
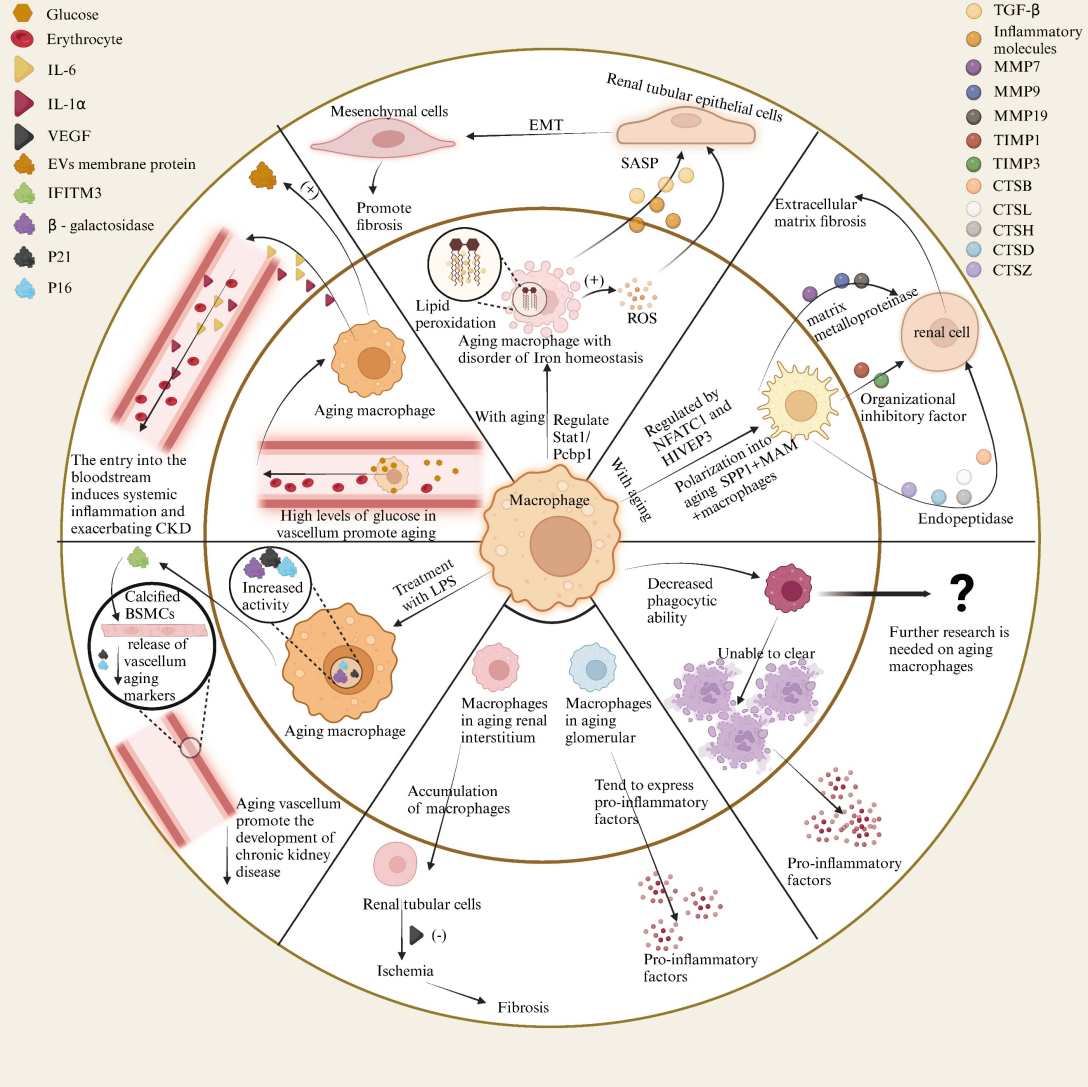
Mechanism of senescence-associated macrophages on renal disease.

Lastly, studies have found that decreased phagocytic function in macrophages may lead to senescence-associated chronic inflammation. This might be related to the continuous release of pro-inflammatory factors by unphagocytosed apoptotic tissue. Although the study did not directly state that senescent macrophages cause senescence-associated chronic inflammation, considering that phagocytic function is a key alteration in senescent macrophages, this mechanism warrants further in-depth research^215^. The mechanism of senescence-associated macrophages on renal disease is shown in Figure 4.

## 5. Senescence-related macrophages as therapeutic targets

Currently, research on therapeutic strategies targeting aging macrophages in the kidneys is limited. However, recent studies on aging macrophages in other tissues have primarily focused on the elimination or improvement of aging macrophages and the modulation or neutralization of SASP-related factors. Here, we provide a brief summary of these studies and offer insights into their potential application in renal research.

### 5.1 Relieving the aging of macrophages

Due to the presence of inflammatory aging and immune aging, the interaction between macrophages and the microenvironment accelerates their aging. Therefore, delaying macrophage aging is a promising therapeutic direction. In the context of diabetes-induced hyperglycemia, where both macrophages and endothelial cells undergo aging, SOD-1 has been shown to significantly reduce oxidative stress, thereby mitigating endothelial cell aging. Although the role of SOD-1 in macrophages has not been thoroughly explored, it warrants further investigation^224^. Additionally, targeting the inhibition of CXCR2 chemokines within the SASP has been found to promote epithelialization of epidermal wounds and alleviate macrophage aging^142^. Salvianolic acid B (SAB) is a water-soluble polyphenolic compound extracted from Salvia lactiflora. In a study on aging lung macrophages, SAB was found to prevent lung fibrosis and cellular senescence in mice by inhibiting the binding of transcription factor SP1 to the SASP promoters (P21 and P16). It also reversed the senescence phenotype of lung macrophages and reduced the activation of pulmonary fibroblasts, highlighting SAB as a potential therapeutic agent for aging-related macrophages^225^.

#### 5.2 Clearance of senescent macrophages

In addition to delaying cellular aging, clearing existing senescent macrophages is also a strategy. Senolytic agents such as navitoclax and the combination of dasatinib and quercetin (D&Q) have demonstrated selective clearance of senescent cells, reducing pulmonary disease and inflammatory responses associated with SARS-CoV-2 infection. This suggests that senolytic therapy has potential applications in the treatment of COVID-19 and other viral infections^148^. Moreover, D&Q has been shown to decrease the burden of senescent cells, including CD+68 macrophages, in the adipose tissue of human subjects with diabetic nephropathy, as well as reduce plasma SASP factors, though the effects were less pronounced in skeletal muscle and liver^226^, ^227^. In Duchenne muscular dystrophy, fisetin has been shown to reduce the number of aging macrophages and decrease SASP factor levels, thereby improving muscle stem cell proliferation and reducing fibrosis[144]Metformin, extensively studied in the context of diabetic nephropathy, exhibits anti-inflammatory and antioxidant properties, enhances autophagy, and prevents fibrosis. It has been shown to reduce the number of ovarian macrophages and increase the number of B cells, T cells, and mast cells in aged mice, thereby alleviating fibrosis to some extent^228^.

#### 5.3 Restore the function of senescent macrophages

Restoring the diminished functions of senescent macrophages is a more conservative therapeutic approach in situations where clearing senescent macrophages or delaying their aging is not feasible. Studies have found that inhibiting ADAM17 in the liver can reduce the cleavage of MerTK, thereby restoring the phagocytic function of aged macrophages. Furthermore, ROS has been shown to promote the activation of ADAM17, which, to some extent, suppresses the phagocytic function of aged macrophages. Therefore, controlling ROS levels can also restore the phagocytic activity of aged macrophages^229^. In another study, allicin was found not only to reduce the expression of p16 and p21 in macrophages but also to decrease the ubiquitination of Sestrin2 by inhibiting RBX1 expression, thereby further enhancing autophagic activity in macrophages and suppressing aging^102^.

#### 5.4 Gene therapy

Gene therapy is an emerging therapeutic approach that is gradually being developed. Studies have shown that in macrophages, caspase-5 (in humans) promotes IL-1α cleavage and drives SASP production^230^. This provides a potential strategy for targeting IL-1α to inhibit the secretion of SASP by aging macrophages. BDR4 is another potential therapeutic target. Research indicates that aging macrophages, through the activation of NF-κB, increase BDR4 expression, which is associated with enhanced lipid accumulation and the aging phenotype. BDR4 is a key factor in regulating the inflammatory and senescence-related chromatin environment in aging macrophages, and modulating it could effectively improve atherosclerosis. Given that atherosclerosis is a factor in chronic kidney disease, treating atherosclerosis could potentially improve chronic kidney disease outcomes^231^. Additionally, aging macrophages can secrete EVs, which contain specific proteins, mRNAs, and miRNAs that interact with the NF-κB pathway, potentially reducing senescence in cancer cells. Whether this mechanism can be applied to other cells for anti-aging purposes requires further research^139^.

#### 5.5 Advantages of Combining Multiple Therapeutic Approaches

When addressing complex aging-related diseases, single therapeutic strategies often have limitations, whereas a combination of multiple approaches can produce synergistic effects. For instance, combining methods to delay aging, clear senescent cells, and restore function can simultaneously suppress aging processes, reduce inflammatory burden, and partially restore tissue functionality. The addition of gene therapy provides technical support for precise regulation, further enhancing therapeutic efficacy. By integrating these strategies, it is possible to not only improve effectiveness and extend the duration of disease control but also potentially reduce drug dosages and side effects, making treatment more comprehensive and lasting.

## 6. Summary

This review presents the origin of macrophages, their polarization process, and their functional changes under conditions of aging and inflammation. It also highlights the critical roles that macrophages play in pathological processes such as kidney disease, fibrosis, and tumors. However, research on the transitions and roles of different subtypes of senescent macrophages in kidney disease, as well as their heterogeneity in different kidney regions, remains limited, especially with regard to specific signaling pathways. Future research should focus on the dynamic changes of senescent macrophages under different renal pathological conditions, especially the interaction mechanisms between senescent macrophages and other cell types in the kidney. By studying these mechanisms in depth, we can better understand the functional changes of macrophages under various pathological conditions and thus provide a theoretical basis for the development of new therapeutic strategies. In addition, as technology advances, future research can utilize cutting-edge techniques such as single-cell sequencing and spatial transcriptomics to further elucidate the molecular mechanisms and cellular heterogeneity of aged macrophages in disease progression.

## Figures and Tables

**Table 1:** Markers of senescent macrophages.

Markers of senescent macrophages	Types of macrophages	Joint indicators	References
LYVE1	Skeletal muscle macrophages in C57BL/6JN mice; Isolated skeletal muscle macrophages from C57BL/6JN mice *in vitro*; Macrophages from the hind paws of Lepr db/db diabetic mice.	Low expression of MHC II;Low expression of PRMT5.	184-188
GCA	Mouse bone marrow macrophages (intracallus macrophages).	Aging of the SSPC.	189,190
CD22	Microglia from CX3CR1-GFP mice;iPSC-derived macrophages.	SBMP/TGF-β signaling pathway (upregulation of genes such as SREBF1, FOXC1, BMP4);Activation of ITIMs downstream signaling.	183,191
GLUT1	Skeletal muscle macrophages in C57BL/6 mice.	Glycolytic enzymes (downregulation of genes such as Aldoa, Eno, Gapdh, Gpi-1, Hk2, Ldha, Pfkp, Pgam1, Pgk, Pkm, Slc2a1, Tpi-1);Impaired HIF-1α transcription.	192
CD38	F4/80-positive macrophages derived from the spleen of C57BL/6 mice.	High expression of SA-β-Gal;High expression of p16;High expression of p21;High expression of Arg1.	182
Arg1	F4/80-positive macrophages derived from the spleen of C57BL/6 mice.	High expression of SA-β-Gal; High expression of p16; High expression of p21; High expression of CD38.	182
CD9	C57BL/6J mice and ffat samples from epididymal adipose tissue.	High expression of SA-β-Gal; High expression of p16; High expression of p21.	168
CD206	ApoE-/- mice and Ldlr-/- mice, vascular tissues from human spleen and testis.	High expression of SA-β-Gal; High expression of p16; High expression of p21; High expression of CD9.	169
TREM2	5×FAD mouse model of amyloidosis.		170
Gpnmb	C57BL/6JN mice and CD11b+macrophage	High expression of Fabp4/5; High expression of Gsr; High expression of Hp; High expression of Prdx1/5/6; High expression of S100a8/ 9 and IL-1β.	171
